# From kitchen to lab: fabricating Ge nanoparticles using a household blender

**DOI:** 10.1039/d6na00584e

**Published:** 2026-10-09

**Authors:** Olha Pylypova, Ivan Lysenko, Kostyantyn Paliienko, Alexander Zaderko, Valeriy Skryshevsky, Vladimir Lysenko

**Affiliations:** a Educational and Scientific Institute of High Technologies, Taras Shevchenko National University of Kyiv 01601 Kyiv Ukraine olha.pylypova@knu.ua; b Ingénierie des Matériaux Polymères, UMR CNRS 5223, Université Claude Bernard Lyon 1 69622 Villeurbanne cedex France; c Universite Lyon 1, CNRS, Institut Lumière Matière, UMR5306 F-69100 Villeurbanne France

## Abstract

In this work, a simple and accessible method for producing colloidal Ge nanoparticles (GeNPs) in ethanol medium using an ordinary kitchen blender is demonstrated. The obtained GeNPs exhibit a polydisperse size distribution in the range of 20–200 nm with characteristic maxima near 30 nm and form stable colloidal solutions with *Z*-potential values of around −20 ± 8 mV. Morphological and spectroscopic studies confirmed the formation of partially oxidized crystalline GeNPs with pronounced nanoscale effects. The UV-Vis absorption spectra of the GeNPs demonstrate a broad asymmetric peak with a maximum near 4.4 eV. Spectral deconvolution revealed two overlapping components at 4.25 eV and 4.5 eV, which are associated with the E_2_ interband transitions in crystalline Ge and their modification in nanoparticles under the influence of quantum confinement effects and surface oxidation. The PL/PLE spectra reveal a dominant blue-green photoluminescence in the 430–520 nm range under UV excitation (250–395 nm), which is attributed to a combination of quantum confinement in the GeNPs and radiative recombination through defect states and oxygen-containing centers at the Ge/GeO_*x*_ interface.

## Abstract 2

Effortless and low-cost transformation of bulk germanium into nanoparticles using an ordinary kitchen blender (image created with AI).

## Graphical abstract

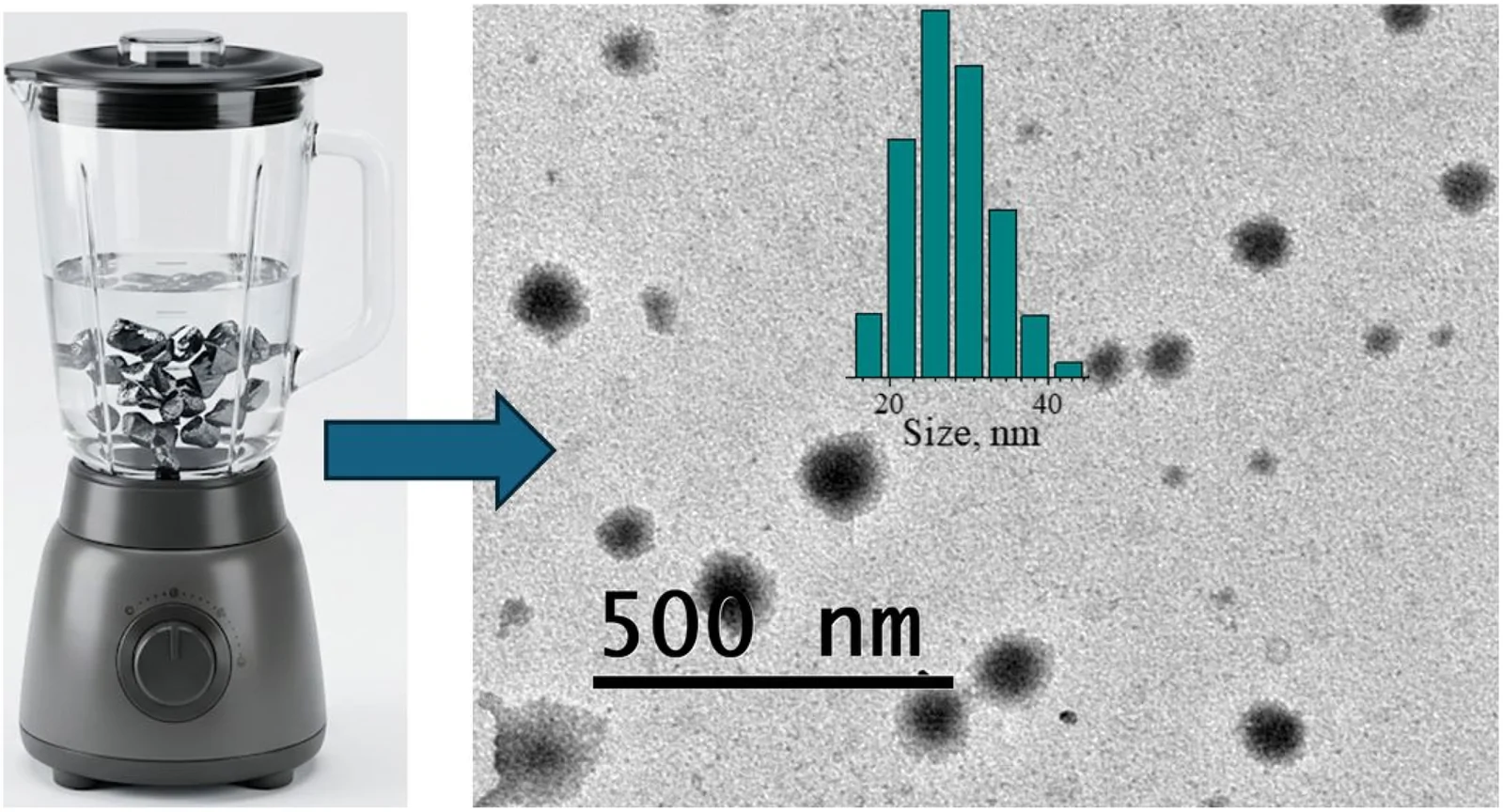

In the pursuit of sustainable, scalable, and low-cost nanotechnology, scientists often turn to sophisticated equipment, such as high-energy ball mills,^1^ laser ablation set-ups,^2^ chemical vapor deposition reactors,^3^*etc.* Recent research has demonstrated that a common kitchen blender can serve as a powerful tool for producing high-quality nanomaterials.^4^ One of the first and most groundbreaking applications of kitchen blenders in nanotechnology is the exfoliation of graphene.^5^ For example, in a study by Varrla *et al.*,^6^ a household blender was used to exfoliate graphite flakes in an aqueous surfactant solution, producing defect-free graphene sheets with concentrations reaching 1 mg mL^−1^ and production rates of 0.15 g h^−1^. This method outperforms traditional sonication techniques, which often suffer from low scalability and high energy consumption.

Another recent remarkable application of kitchen blenders has been in the synthesis of silicon nanoparticles (SiNPs). In research by Chaâbani *et al.*,^7^ a blender was used to grind bulk silicon lumps in deionized water, producing colloidal SiNPs with diameters ranging from 100 to 200 nm. These nanoparticles exhibit Mie resonances enabling applications in structural coloration, nanoantennas, and optical sensors. By harnessing the sheer mechanical force of a kitchen blender, researchers can transform bulk silicon lumps (or wafers) into high-value SiNPs with extraordinary potential in various fields.^8^ Thus, unlocking low-cost mass production of SiNPs seems to lie not in a multimillion-dollar cleanroom, but in the humblest of household appliances used to make smoothies. Traditional methods for SiNP synthesis, such as (electro-)chemical etching^9,10^ or laser ablation,^11–13^ are often expensive, energy-intensive, and limited in scalability. In contrast, the blender method is rapid and easily accessible, democratizing nanotechnology for labs with limited resources. Moreover, the ability to recycle silicon waste (*e.g.*, discarded wafers or solar cell scraps) into functional nanoparticles aligns perfectly with the global push for a circular economy. However, as with any disruptive technique, challenges remain. Particle size distribution, surface oxidation, and batch consistency must be carefully controlled to unlock the full potential of blender-synthesized SiNPs.

But why a blender? The answer lies in its brutal efficiency. A blender's razor-sharp blades, spinning at thousands of revolutions per minute, generate intense shear forces and cavitation and thus create conditions that rival those of traditional top-down nanofabrication methods. When combined with a suitable liquid medium (*e.g.*, ethanol, water, or even cooking oil), this everyday appliance becomes a factory of nanoparticles with tunable sizes, high surface areas, and minimal contamination.

In this article, we describe application of a kitchen blender for fabrication of luminescent Ge nanoparticles (GeNPs). We'll show that this unconventional yet brilliantly simple technological approach allows fabrication of GeNPs from bulk Ge substrates.

GeNPs are similar to SiNPs in terms of physico-chemical properties.^14^ The interest in GeNPs is driven by their size-dependent optical and electronic properties and their potential applications in optoelectronics, photonics, biological imaging, therapeutics, and energy-storage devices.^15,16^ In particular, Ge nanomaterials are attractive because of their strong optical absorption, high carrier mobility, narrow bulk band gap, and the possibility of tuning their optical properties through particle size and surface chemistry. These characteristics make GeNPs promising for a wide range of applications, including photodetectors, photovoltaic devices, bioimaging, and lithium-ion batteries.^17^ Strong light absorption by GeNPs in the visible-near IR range makes them excellent candidates as sensitizers of photo-induced hyperthermia.^15^ Being an indirect gap (0.67 eV) semiconductor, it is difficult to fabricate red-IR luminescent GeNPs. Usually, photoluminescence of GeNPs corresponds to the blue spectral region.^16,17^

GeNPs were already reported to be synthesized using a variety of methods. Bottom-up methods, such as chemical vapor deposition (CVD) or laser pyrolysis, enable the growth of Ge nanoparticles from gaseous precursors like GeH_4_^18^ and Ag-catalyzed CVD^19^ allowing control over crystallinity, localization and size. Additionally, wet-chemical synthesis routes provide scalable production of Ge nanoparticles with tunable optical and electronic properties.^20–23^ Top-down approaches include mechanical milling to produce nanoparticles with controlled sizes^24,25^ and laser ablation in liquids, which generates high-purity Ge nanoparticles by ablating bulk germanium in a liquid medium.^26,27^

In this work, an n-type (Sb-doped) (100)-oriented monocrystalline 4-inch Ge wafer with electrical resistivity in the range of 0.005–0.02 Ω cm purchased from BT Electronics was milled in 370 mL of pure ethanol by means of a kitchen blender (ProBlend 6, Philips). No surfactants were used in this work. Sharpening of the blades can be performed before the grinding to ensure the smallest diameters of GeNPs. Such blenders are not designed for continuous operation at high speeds for long times due to excess heating. To reduce the increase in temperature and pressure in the glass jar caused by blending at higher revolution speed, the mixer was turned off for 2 min after every 3 min of blending. 20 grams of Ge fragments were weighed and added to the jug. Then 370 mL of ethanol was also poured into the jug. The grinding process was carried out in repeated cycles of 3 min of blending followed by 2 min of cooling, with a total processing time of 60 min, including the cooling intervals, to obtain a dispersion of GeNPs. After sedimentation, the upper 75% of the dispersion was carefully collected to minimize the contribution of larger, rapidly sedimenting particles and to obtain a fraction enriched in smaller GeNPs. This process is illustrated in the scheme shown in Fig. 1.

**Fig. 1:**
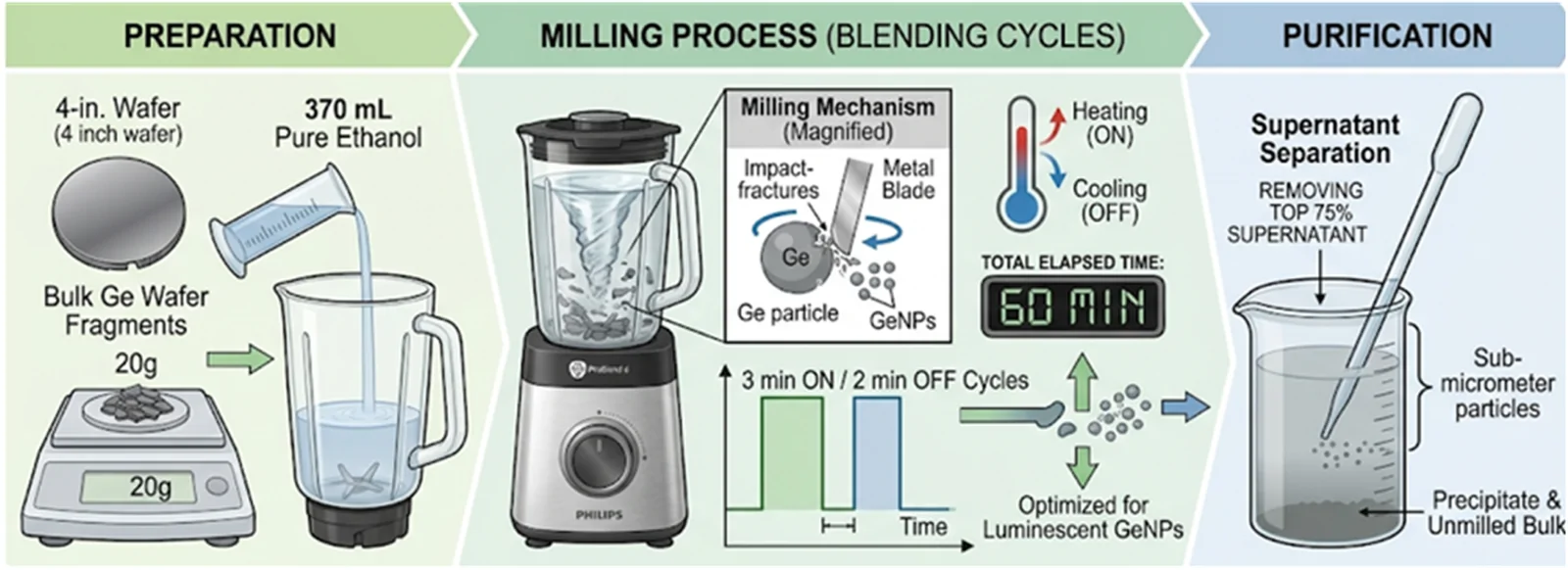
Blending process of monocrystalline Ge substrates (image created with AI).

To examine morphology and structural characteristics of the synthesized GeNPs, transmission electron microscopy (TEM) analysis was carried out. TEM images were obtained at the Microstructures Technological Center (CTµ, Villeurbanne, France) using a TEM microscope JEOL 1400 Flash (accelerating voltage of 120 kV, LaB6 cathode) equipped with a Gatan RIO16 camera (4K × 4K). The sample was fixed on a 200 mesh copper grid with carbon coating (CF200-CU-50, Electron Microscopy Sciences™) in the following way: 0.3 µL of stock solution was dropped on the grid surface and dried for 20 min at 20 °C. Fig. 2a presents a TEM image of GeNPs collected from fresh colloidal solutions. A large amount of small spherical NPs can be observed. The inset in Fig. 2a shows individual Ge particles with a dark crystalline core surrounded by a lighter oxide shell. This structure is due to the formation of a thin layer of germanium oxide (GeO_*x*_) on the surface of the nanoparticles. Size distribution of the GeNPs was deduced by calculation of about 359 NPs from TEM images. Diameter values of GeNPs range from 10.0 nm to 70.0 nm according to the size distribution histogram shown in Fig. 2b.

**Fig. 2:**
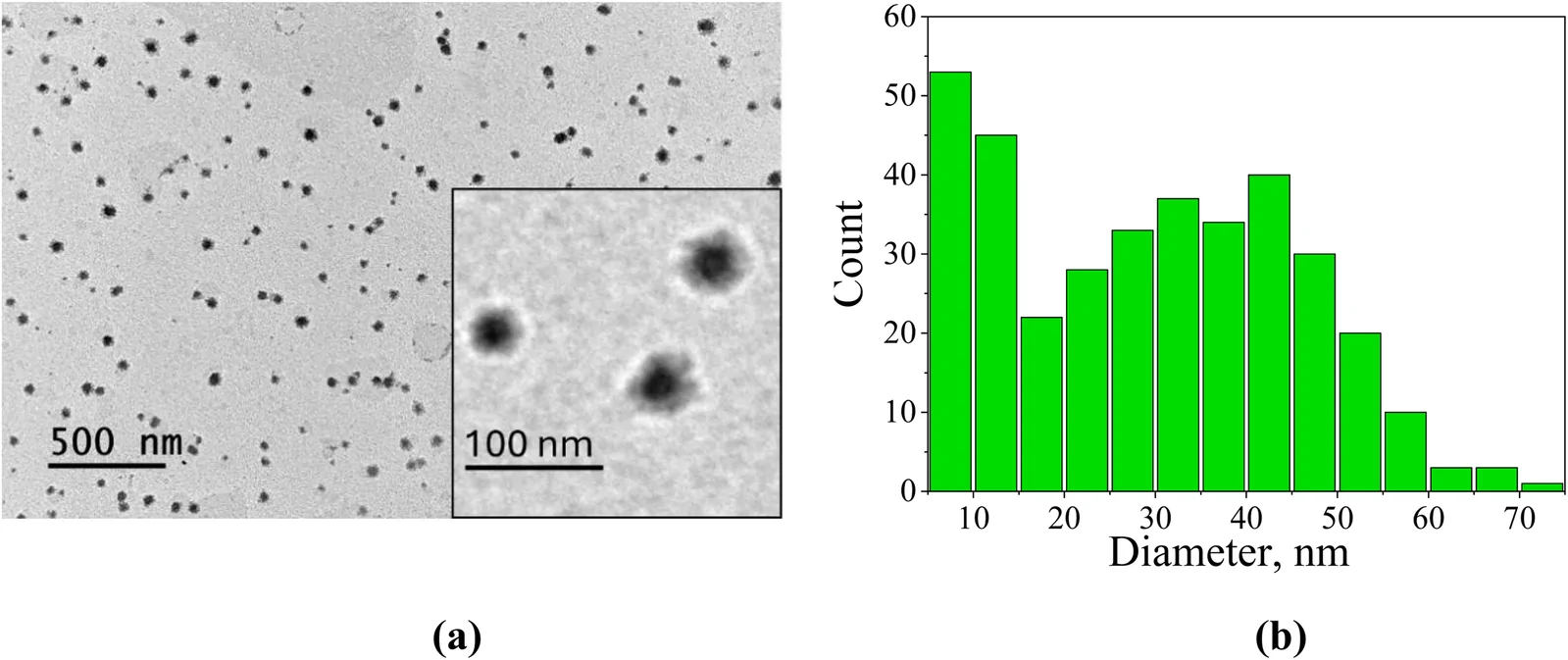
(a) TEM image of as-prepared GeNPs; inset: the TEM image of a single GeNP; (b) size distribution of GeNPs deduced from the TEM picture.

Macro-scale dynamic light scattering (DLS) measurements of size distribution were performed on diluted colloidal solutions of the GeNPs using Zetasizer Nano ZS equipment from Malvern Instruments (He–Ne laser 633 nm, 5 mW, with a 173° Non-Invasive Back Scatter (NIBS) detector and narrow band filter). For the DLS measurements, the initial ethanol dispersion of GeNPs was mixed with deionized water at 1 : 1 (v/v). For the processing of the DLS data with the Zetasizer software, dispersion medium parameters corresponding to a 1 : 1 ethanol/water mixture were used. In addition, the appropriate viscosity value of the mixture (2.4 mPa s for the ethanol/water mixture at 25 °C) was also used.

Size distribution of freshly prepared GeNP colloids covers the range of 20–200 nm with two pronounced maxima at 30 nm and 90 nm (see Fig. 3a). The first maximum is consistent with the size range of individual nanoparticles observed in the TEM images (Fig. 2). The second maximum, centered at approximately 90 nm, is most likely associated with transient agglomerates of GeNPs formed in the fresh colloidal dispersion. Such agglomerates may result from interparticle van der Waals interactions and interactions between the partially oxidized surfaces of GeNPs. Additional DLS measurements, conducted after aging the dispersion for 3 months, showed that the initially bimodal distribution becomes monomodal over time and the population with the larger hydrodynamic size disappears (Fig. 3c). This indicates that the second population is most likely associated with unstable agglomerates, rather than a separate fraction of large primary GeNPs. Such temporal evolution may be caused by a change in the surface state of the GeNPs in the water–ethanol medium, particularly by the partial dissolution or transformation of surface GeO_*x*_, which alters interparticle interactions and reduces the stability of the agglomerates. The initial fresh GeNP dispersion exhibits moderate zeta potential values of approximately −20 ± 8 mV (Fig. 3b), which suggest partial electrostatic stabilization and are associated with the negatively charged oxide layer on the particle surfaces.^28^ After 3 months of aging, the hydrodynamic size distribution became predominantly monomodal, and the zeta potential distribution became more symmetric and closer to a Gaussian-like shape (Fig. 3d).

**Fig. 3:**
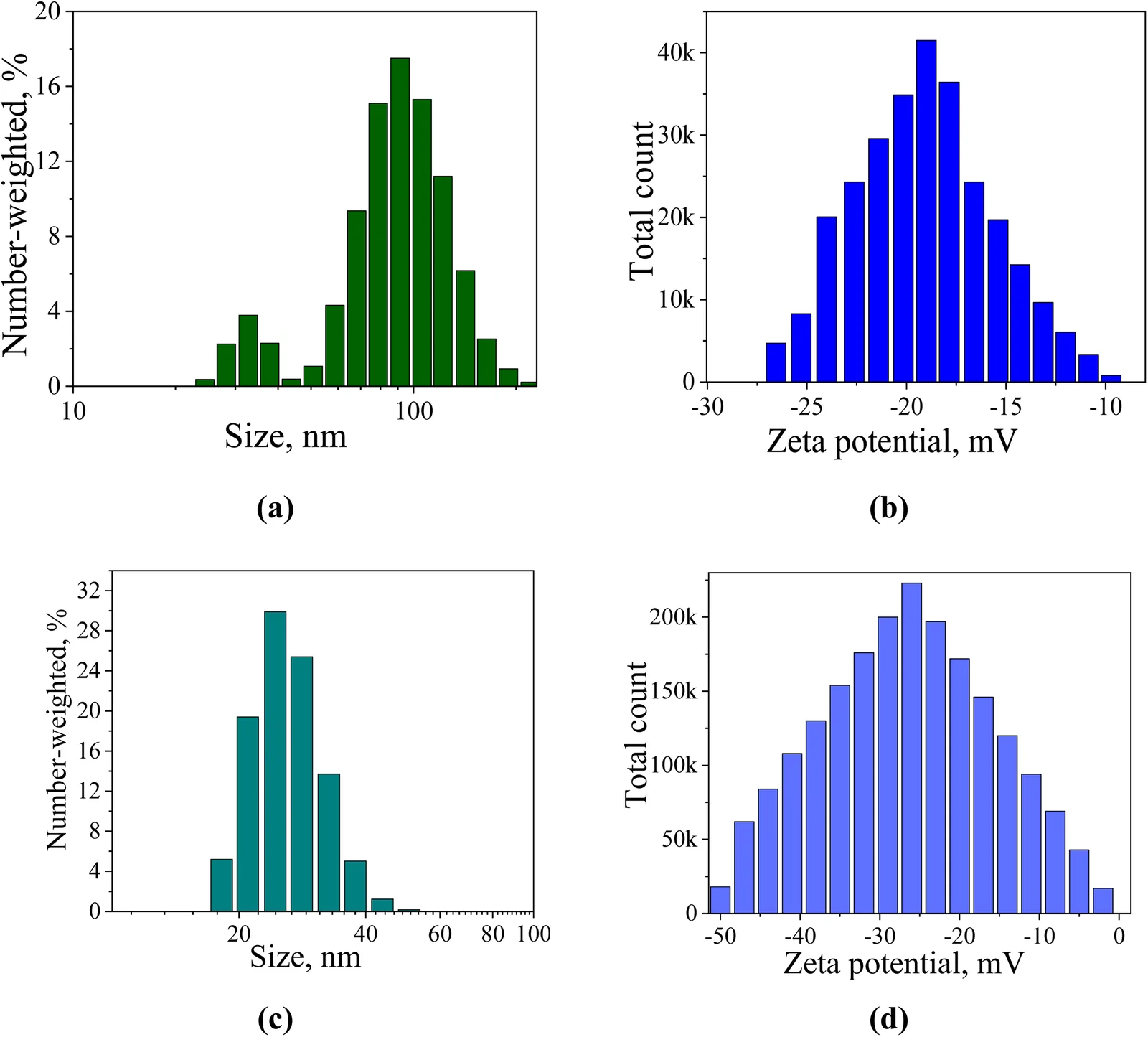
(a) Initial size distribution and (b) *Z*-potential distribution of freshly prepared GeNPs at pH 7.34; (c) size distribution and (d) *Z*-potential distribution of GeNPs after three months of storage at pH 7.34.

The presence of a GeO_*x*_ shell on the surface of GeNPs is further supported by the FTIR spectrum shown in Fig. 4a, which provides additional information on the surface chemistry of the GeNPs. FTIR (Fourier-transform infrared) spectra of the GeNPssamples were acquired on an IRAffinity-1, Shimadzu®, in attenuated total reflection (ATR) mode, using a PIKE MIRacle™ ATR platform (diamond crystal plate). Dried amorphous powders of the lyophilized samples were placed on a crystal plate, and spectra in a 500–4000 cm^−1^ range with a resolution of 0.5 cm^−1^ were recorded and analyzed with a Shimadzu IR solution v1.50®. Two intense n(Ge–O) overlapped bands can be observed at 566 and 680 cm^−1^. They correspond, respectively, to bending and stretching vibration modes of the Ge–O–Ge bonds.^29^ A much weaker band at around 1644 cm^−1^ is due to bending of H–O–H bonds. Two spectral bands at 1900 and 2100 cm^−1^ may be attributed to the stretching vibration modes of the Ge–H bonds.^30,31^ It has been shown in the literature that ethanol can dissociatively adsorb onto the Ge surface through O–H bond cleavage, forming surface Ge–H and Ge–OCH_2_CH_3_ groups.^30^ Thus, ethanol can act as a potential source of hydrogen for the partial H-termination of the freshly formed Ge surface. The bands in the region of approximately 1996–2100 cm^−1^ fall within the spectral range characteristic of Ge–H stretching modes. However, since a direct analysis of surface H-termination was not performed in this study, this assignment should be considered tentative rather than definitively established.

**Fig. 4:**
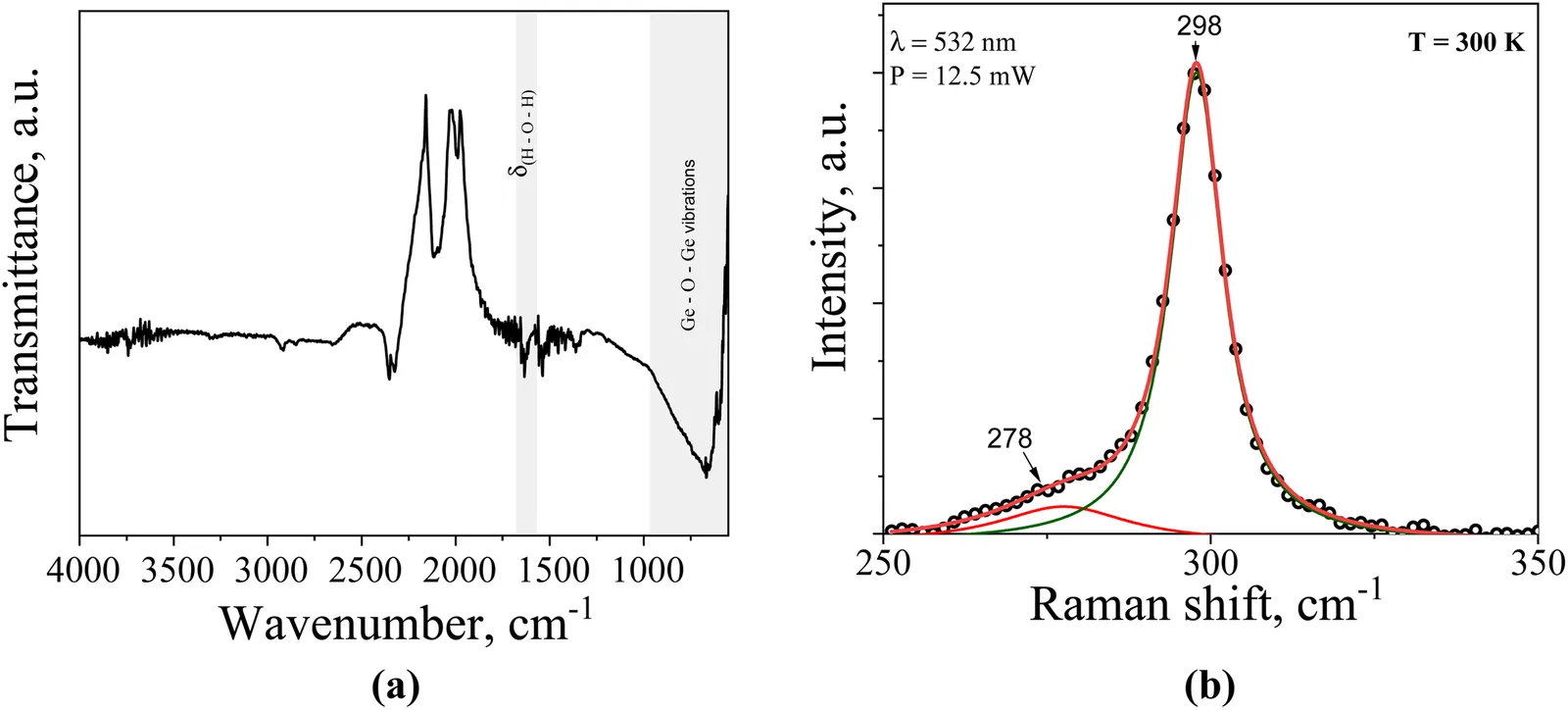
(a) FTIR spectrum of GeNPs after baseline correction and (b) Raman spectrum of the initial Ge nano-powder obtained by blending.

The typical Raman spectrum of the GeNPs shown in Fig. 4b and recorded at room temperature (*T* = 300 K) using Novations RMS2 equipment under excitation at *λ* = 532 nm with a laser power of *P* = 12.5 mW exhibits the characteristic signatures of a nanocrystalline structure accompanied by a disordered phase. The laser beam was focused down to a spot diameter of 1.5 µm on the surface of Ge nano-powders with a 50× magnification and 0.45 numerical aperture microscope objective. The primary optical phonon peak is centered at 298 cm^−1^, which is noticeably shifted toward lower energies (lower wavenumbers) compared to bulk monocrystalline Ge (∼300–302 cm^−1^). This downshift, along with the slightly asymmetric peak shape that extends into the lower-energy region, is driven by the spatial phonon confinement effect within the nanoscale crystalline Ge cores.^32^ Furthermore, spectral deconvolution (depicted by the solid fitting lines) reveals an additional broad band centered near 278 cm^−1^, which corresponds to the transverse optical (TO) mode of the amorphous Ge phase (a-Ge).^32^ The presence of this prominent amorphous component confirms the formation of a disordered or partially oxidized shell surrounding the crystalline cores during the intensive mechanical milling process.

UV-visible (UV-Vis) absorbance spectra of the GeNP-based colloidal solutions were collected in the 200–800 nm wavelength range at room temperature (20 °C) using a Cary UV-Vis spectrophotometer (Agilent Technologies, Mulgrave, Australia). The UV-Vis absorption spectrum of GeNP-based colloidal solutions shown in Fig. 5a reveals characteristic electronic transitions of the GeNPs. Black points correspond to the original UV-Vis absorption spectra. After subtraction of exponential-like baselines (see the red line in the insets), one can get a complex spectrum with some visible spectral features.

**Fig. 5:**
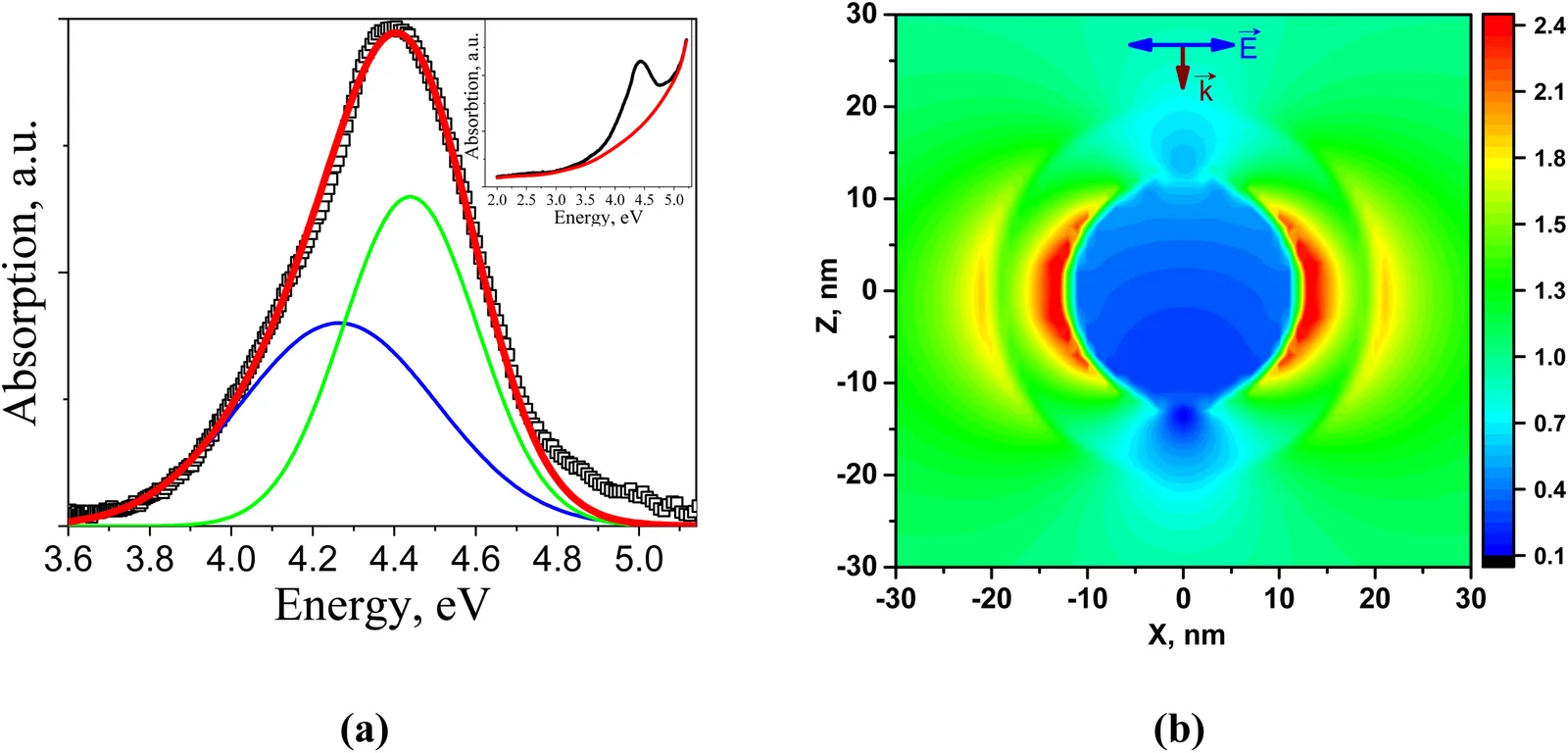
(a) UV-Vis absorbance spectra of GeNPs. (b) Simulated distribution of the photoinduced local electric field around a single GeNP with a 24 nm diameter and 8 nm thick GeO_2_ shell at an absorption maximum energy of 4.4 eV (280 nm).

The main visible peak on the absorbance spectrum shows a broad asymmetric peak globally centered at 4.4 eV. To analyze fine spectral features, spectral deconvolution of the UV-Vis absorption spectrum into elementary Gaussian-like peaks was performed. As shown in Fig. 5a, two characteristic overlapping peaks can be identified at 4.25 eV and 4.5 eV. The first peak may correspond to the well-known most intense spectral feature of the imaginary dielectric function (E_2_) in bulk crystalline Ge.^33^ It primarily arises from electronic transitions at the *X* symmetry point and along the S direction of the Brillouin zone.^33^ Involving a large volume of the Brillouin zone and leading to a massive joint density of states, the E_2_ peak is thus the dominant absorption feature in the ultraviolet range. When bulk Ge is reduced to nanoparticles, the E_2_ transition energy can be changed. For example, an increase in the energy gap between the valence and conduction states due to the quantum confinement effect in small GeNPs causes the E_2_ peak to blue-shift.^34^ Therefore, the second peak centered at 4.5 eV can correspond to E_2_ electronic transitions in relatively small GeNPs. In addition, because fabrication methods like mechanical milling produce a large distribution of particle sizes and shapes, the E_2_ peak appears significantly broader and less sharp than in a perfect bulk crystal. Finally, mechanical milling can introduce significant internal strain, point defects and interruption of crystal translation symmetry at the Ge/GeO_*x*_ interface with subsequent relaxation of the k-selection rules in partially oxidized GeNPs. All these mechanisms perturb the local potential with further broadening of the E_2_ electronic transition.^35^ Moreover, bandgap of the non-stoichiometric Ge oxide (GeO_*x*_) covering crystalline GeNPs is characterized by the value 4.3 ± 0.2 eV which is significantly lower than that of stoichiometric GeO_2_: 5.4–5.9 eV.^36^

The finite-difference time-domain (FDTD) module of the Lumerical software package^37^ solving Maxwell's equations was used to calculate the 2D spatial distribution of the photoinduced local electric field around a single Ge/GeO_2_ NP (Fig. 5b) under excitation at an absorption maximum of 4.4 eV (280 nm) as shown in Fig. 5a. The simulation model consisted of a spherical GeNP with a 24 nm diameter and 8 nm thick GeO_2_ shell in ethanol and a plane wave source based on a total-field scattered-field technique surrounded by perfectly matched layer boundaries. The refractive indices of Ge, GeO_2_ and ethanol were taken, respectively, from the Lumerical database of materials and online refractive index database.^38^ The used mesh step was 1 nm. The incident electromagnetic wave is depicted at the top center, with its electric field vector (*E⃑*) polarized horizontally along the *X*-axis and its wave vector (*k⃑*) propagating downwards along the *Z*-axis. The accompanying vertical color bar maps the relative electric field intensity, ranging from a minimum of 0.1 (dark blue) to a peak of 2.4 (red). The interior of the Ge core appears predominantly dark blue, indicating a suppression of the internal field, whereas the electric field enhancement maximum emerges symmetrically on the outer lateral boundaries of the GeO_2_ shell, aligning precisely with the horizontal polarization direction of the incident electric field. Conversely, regions of reduced intensity are observed at the top and bottom poles along the propagation axis, altogether characterizing a distinct dipole-like resonance pattern characteristic of such nanostructures under plane wave excitation. The localized field enhancement (reaching up to a factor of 2.4) is concentrated specifically within the 8 nm GeO_2_ shell and at the interfaces. This demonstrates that the dielectric oxide layer effectively acts as an optical nano-antenna, trapping and amplifying electromagnetic energy at the particle's periphery rather than in the bulk.

To investigate the optical emission properties of the GeNPs, fluorescence measurements were performed with a Cary Eclipse fluorescence spectrophotometer (Agilent Technologies, Les Ulis, France) equipped with a 75 kW xenon flash lamp (pulse width of 2 µs and a 100 Hz repetition rate). Fluorescence maps were recorded for emission wavelengths between 400 and 600 nm and excitation wavelengths from 250 to 395 nm.

Fig. 6 shows the room temperature photoluminescence emission/excitation (PL/PLE) map for the colloidal GeNPs obtained by blending bulk Ge wafers. The PL/PLE spectra indicate the dominating emission in the blue-green spectral region (430–520 nm) under UV excitation (250–395 nm), unlike bulk Ge, which is a poor light emitter due to its indirect bandgap (0.66 eV). Taking into account size distribution values reported above as well as the chemical structure of the partially oxidized GeNPs, their characteristic blue-green PL arises from the complex interplay between two primary mechanisms already earlier reported in the literature: the quantum confinement effect^39^ and the influence of surface states and oxide defects.^40^ As the Ge core size is reduced below the Bohr exciton radius of approximately 24 nm, a very strong quantum confinement effect causes a widening of the GeNP bandgap and a relaxation of the *k*-selection rule, thus effectively allowing quasi-direct radiative transitions to migrate from the infrared into the visible spectrum. This intrinsic physical change is complemented by the chemistry of the Ge/GeO_*x*_ interface, where the amorphous oxide shell introduces localized electronic states within the bandgap. Specifically, radiative recombination occurs at sites such as oxygen-deficient centers and Ge lone-pair electrons, which ensure the intense emission observed in partially oxidized nanostructures. Because the intense near-field is localized exactly where surface defect states and oxygen-deficient centers reside (see Fig. 5b), the excitation efficiency of these specific radiative centers is maximized. This near-field amplification directly explains why the partially oxidized GeNPs exhibit such strong blue-green photoluminescence under UV excitation.

**Fig. 6:**
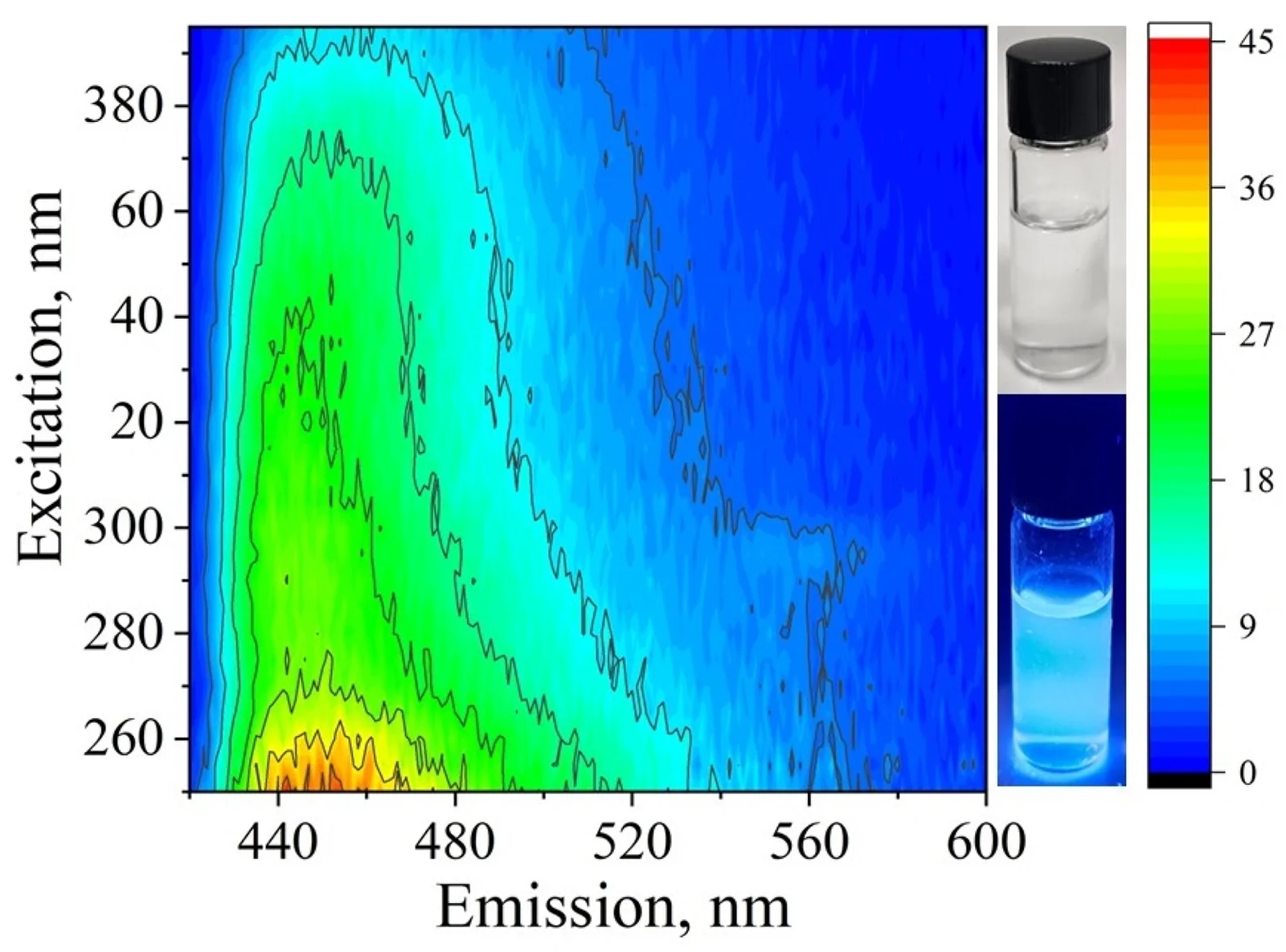
PL/PLE map of GeNP colloids. Insets show colloidal solutions of GeNPs under day light (upper image) and under UV excitation at 360 nm (bottom image).

In summary, this work successfully demonstrates an unconventional, cost-effective and simple top-down approach for the fabrication of colloidal GeNPs using a standard household kitchen blender. By employing an optimized, intermittent grinding-and-cooling cycle in an ethanol/aqueous medium, bulk monocrystalline Ge wafers were efficiently reduced to GeNPs forming stable colloidal solutions with a *Z*-potential of approximately −20 ± 8 mV and a polydisperse size distribution ranging from 20 to 200 nm, featuring a primary size population centered near 30 nm. Comprehensive spectroscopic and optical characterization confirmed that the mechanical shear forces yield crystalline GeNPs subject to partial surface oxidation. The UV-Vis absorption profiles reveal a prominent, broad asymmetric peak near 4.4 eV. Spectral deconvolution of this feature resolved two overlapping sub-components at 4.25 eV and 4.5 eV, which correspond to the characteristic bulk E_2_ interband transitions and their quantum-confined, oxide-modified counterparts at the nanoscale. Furthermore, under UV excitation (250–395 nm), the partially oxidized GeNPs exhibit intense, dominant blue-green photoluminescence spanning 430–520 nm. This efficient visible emission is verified to originate from a synergistic combination of quantum confinement effects within the core Ge matrix and radiative recombination pathways governed by defect states and oxygen-deficient centers at the engineered Ge/GeO_*x*_ interface. Ultimately, this high-shear liquid-phase exfoliation strategy establishes a highly accessible and scalable paradigm for manufacturing functional, luminescent Ge-based nanomaterials without the need for complex chemical precursors or specialized industrial milling equipment. Investigating the effect of centrifugation conditions on the size distribution and properties of individual GeNP fractions is considered a promising direction for future research.

## Author contributions

O. P., V. S., and V. L. – concept and analysis; K. P., A. Z., and I. L. – investigation; O. P. – original draft preparation; V. L. and V. S. – supervision. The manuscript was written through contributions of all authors. All authors have given approval to the final version of the manuscript.

## Conflicts of interest

The authors declare no competing financial interest.

## Data Availability

The data supporting the findings of this study are presented within the article. Additional supporting data are available on Zenodo at DOI: https://zenodo.org/records/23393214.
